# Persian leopard's (*Panthera pardus saxicolor*) unnatural mortality factors analysis in Iran

**DOI:** 10.1371/journal.pone.0195387

**Published:** 2018-04-25

**Authors:** Morteza Naderi, Azita Farashi, Mehdi Alipour Erdi

**Affiliations:** 1 Department of Environmental Sciences, Faculty of Agriculture and Natural Resources, Arak University, Arak, Iran; 2 Department of Environmental Sciences, Faculty of natural resource and environment, Ferdowsi University of Mashhad, Mashhad, Iran; 3 Environmental planning and management, Graduated Faculty of Environment, University of Tehran, Tehran, Iran; Universidade Federal do Parana, BRAZIL

## Abstract

Due to the relatively low offspring survival rate, surviving adult leopards play a critical role in the species’ viability. The unnatural mortality of leopards, caused by human activities can seriously compromise the species’ long-term population survival. An analysis of spatial distribution and sex ratio of unnatural mortality of 147 recorded Persian leopard (*Panthera pardus saxicolor*) carcasses during a fifteen-year period (from 2000–2015) in Iran indicated that road mortality is the second most frequent cause of unnatural mortality of Persian leopards’ after illegal hunting (or prey poisoning, such as poisoned meat) by villagers, shepherds and military forces. The greatest percent of unnatural mortality events were recorded in the Golestan provinc in the north of Iran and eastern most parts of the Hyrcanian forests. Using distribution models of species, based on road accident locations as species data, we mapped the species’ distribution and critical areas of unnatural mortality of Persian leopard that can be used in prioritizing leopard-human conflicts management. Our results showed that mortality records were significantly higher in non-protected compared to protected areas. Males constituted 65 percent of the records used in the study as males dispersed more widely compared to the females. This imbalance can have severe demographic effects. A large proportion of leopards’ activity, occurrence area, and habitat lies in non-protected areas, which is mirrored by the greater number of unnatural mortality outside protected areas. Most of the incidents were due to human factors, thus management interventions such as traffic speed limitations, signs, cameras, and faunal bridges as well as increasing public participation and awareness (especially among rural communities) will positively affect the species’ conservation programs. This research aimed to produce unnatural mortality of leopards’ risk map throughout Iran and discuss the different aspects of this phenomenon, major human-caused threats and the efficiency of the legal protected areas in satisfying the species’ ecological requirements. We propose management interventions such as traffic speed limitations, signs, cameras, and faunal bridges as well as increasing public awareness and participation, especially among rural communities, to support the species’ conservation.

## Introduction

Probabilistic factors such as demography, genetics and environmental stochasticity have been identified as important factors in wild animals’ extinction [1]. However, deterministic factors such as illegal hunting, prey poisoning [2] and road mortality [3] may have a larger role in the eradication of large carnivores, especially big cats [4–7]. Leopards (*Panthera pardus*) are the most widely distributed wildcat, occupying different habitats, from deep forests to steppe deserts and from the fringes of rural areas to remote mountain ranges [8–9]. Globally, more than 65 percent of the endangered Persian leopard’s (*Panthera pardus saxicolor* Pocock, 1927) populations are distributed in Iran [10, 6, 11] but the species’ conservation has faced many challenges. The declining state of Asian Leopards has been reported in many countries. For example, leopards are reported to be likely extirpated in Laos and Vietnam, and nearly extirpated in Cambodia and China while their distribution range has greatly shrunk in Malaysia, Myanmar, and Thailand [12]. Because of their unique habitat usage pattern, occupying a narrow range of environmental conditions, and their special behavioral mechanisms [13], leopards are vulnerable to several types of human-induced factors such as road mortality [6] and hunting related mortality of adults [14]. Due to the relatively low offspring survival rate [15], surviving adult leopards play a critical role in their populations’ viability [16]. As a result, the unnatural mortality of leopards [17] can seriously compromise the species’ long-term population viability [18].

Because investigations of survival rates of leopards and their habitat selection face multiple challenges such as high financial costs involved in population monitoring [19], difficulties in detecting the animals, and their secretive life [20], the optimal sample size cannot be obtained [21, 22]. As an alternative, utilizing the relatively large sample size of unnatural mortality data can provide a useful approach to the species’ conservation in Iran. Swanepoel et al [22] suggested that management interventions and conflict mitigation programs may have the potential to increase leopard survival dramatically by lowering human-caused mortalities outside protected areas.

In this study we aimed 1) to map the Persian leopard's mortality risk map based on collected unnatural mortality, 2) to extract major habitat variables for the unnatural mortality points to infer the macro and meso-habitat selection of the species, 3) to investigate sex ratio among the species unnatural mortality, 4) to discuss the efficiency of the protected areas to satisfy the species’ habitat requirements in the country, and finally, 5) to create initial foundations and persuade authorities to construct faunal bridges especially in the Golestan National park to enhancing the species survival.

## Materials and methods

We collected unnatural mortality data of Persian leopard for 15 years (2000–2015) from most provinces of Iran (S1 Table). These data were obtained from wildlife rangers, non-governmental organizations (NGOs), local authorities and Department of Environment (DOE). From collected data, we listed the cause of mortality, sex, relative age and the location of death.

Fuzzy logic and maximum entropy methods were applied to produce a countrywide unnatural mortality risk map [23]. Fuzzy logic is a broad concept originating from classical logic, which is applicable in computer based decision making processes [24, 25]. Unlike classical logic, which classifies each criterion, false or true (0 and 1), fuzzy logic assigns a value from 0 to 1 to each variable. Based on a membership function each variable in a fuzzy set is computed [26]. In this research, we determined the real value of variables by using a geographic information system (GIS). Then, the value of each corresponding cell for each criterion was extracted based on Persian leopard’s unnatural mortality data. Finally, we extracted all criteria membership functions based the values of maximum of minimum points and plotted a histogram of the point values frequencies. Lastly, we produced the Persian leopard mortality risk map using “AND” and “AR” logics. Matlab was used to produce the eco-geographical fuzzy maps for all variables, which were then overlaid using ArcMap 9.3.

MaxEnt is a general-purpose machine-learning method based on the maximum entropy theory that has been further developed in statistical mechanics for species distribution modeling [27]. The idea behind MaxEnt is to estimates niches by finding the distribution of probabilities closest to uniform (maximum entropy). MaxEnt is subjected to the constraints of feature values matching their empirical average. The importance of environmental variables is evaluated using Jackknife tests [28]. Ten random partitions were used to assess the average behavior of the models [27]. Each partition was generated by cross validation of 75% species occurrence as calibration data, and the remaining 25% as evaluation data. The area under the ROC (receiver operating characteristic) curve, or AUC, was used to evaluate model performance. AUC is a measure of model performance that varies from 0 to 1 [29]. An AUC of 0.50 indicates that the performance of the model has not been substantially better than random, whereas a value of 1 indicates perfect discrimination [30].

### Environmental predictor variables

Environmental variables included land cover and land use characteristics and topography. Land use and land cover data were obtained from the Iranian Forests, Range and Watershed Management Organization (IFRWO) (http://frw.org.ir). The data was derived from 30m Landsat Enhanced Thematic Mapper Plus (ETM+) imagery from 2005 to 2015. Topographic variables were obtained from a digital elevation model (DEM) that was generated by the National Cartographic Center of Iran (NCC) (http://www.ncc.org.ir) at 1:25000 scale. The multi-collinearity test was conducted using the Pearson correlation coefficient to examine cross-correlation. Variables with a cross-correlation coefficient value greater than ±0.6 were excluded. For all distance variables Euclidean distance analysis in ArcMap 9.3 was used.

## Results

**Fig 1:**
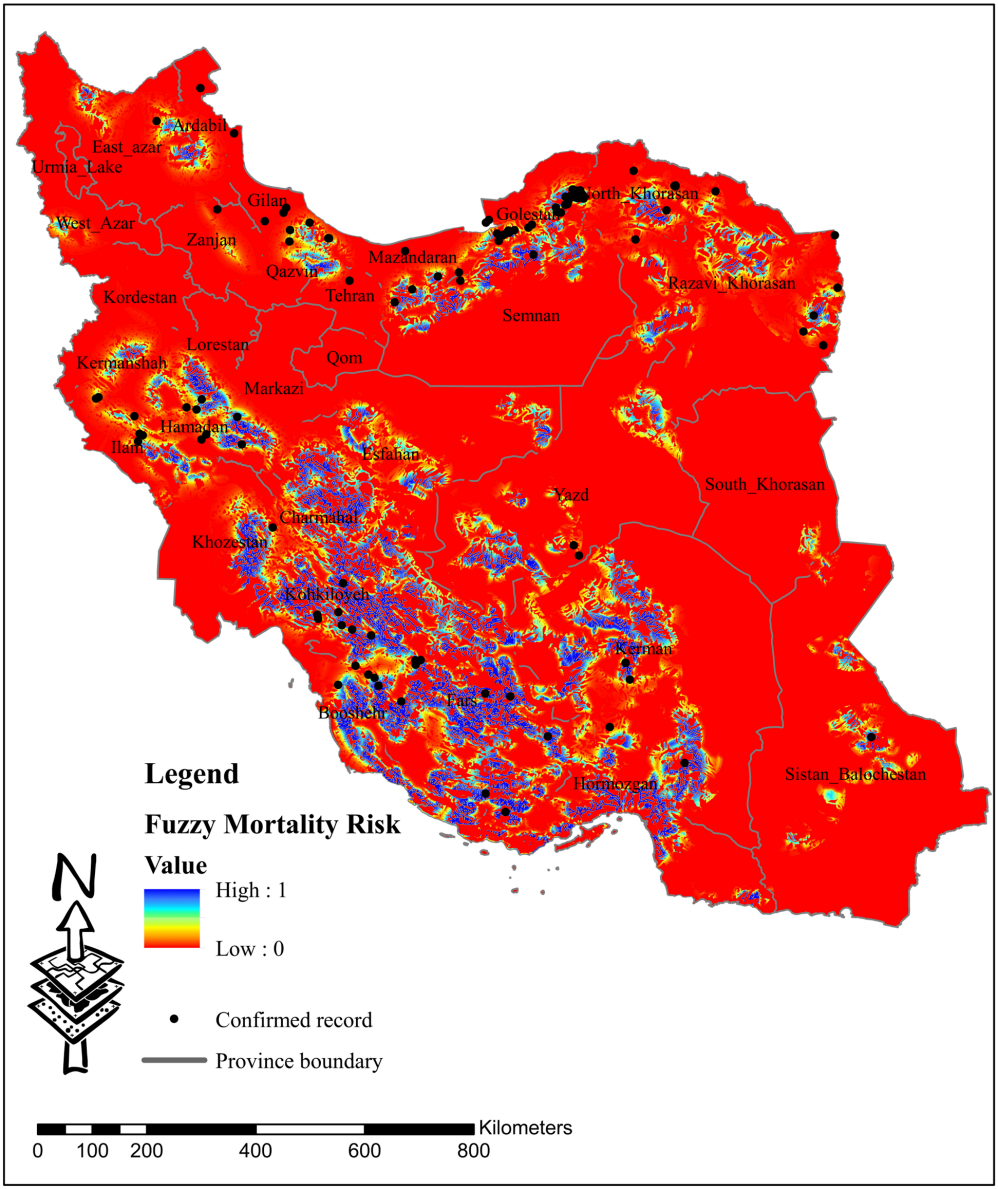
Fuzzy leopard unnatural mortality risk map which shows hot areas facing with unnatural mortality events in red.

A total of 147 unnatural deaths of Persian leopards were recorded from 2000 to 2015 (of which the sex of the case was recorded only for 76 cases in the different databases), with 49 specimens being males and 27 females. Our results indicated that more than 60 percent of unnatural mortality was due to targeted killing by toxic bait, shooting by ranchers, trophy hunters and by military forces. Road mortality was responsible for more than 26 percent of the total Persian leopards’ unnatural mortality during the investigation period (n = 37). About 78 percent (n = 29) of the road kills had happened in Golestan National Park, in particular on the busy Tehran-Mashhad road (Fig 1).

Male leopards were respectively 2.5 and 1.5 times more likely than females to die in collisions with vehicles and by poisoning. Twenty-one cases were attributed to unknown or natural causes such as intraspecific competition and drought, based on the examination of carcasses. More than two-thirds of all cases were recorded outside protected areas.

### Fuzzy unnatural mortality risk map

The unnatural mortality risk map was generated by assigning continuous fuzzy values to each cell in the environmental variables raster maps in Matlab and overlaying the maps in ArcGIS (Fig 1). The resulting map shows two geographical areas with the highest unnatural mortality risk. Golestan National Park located in the northeastern Iran, and parts of the Zagros mountain range in the southwest. Golestan National Park is located in the heart of the Hyrcanian old growth forests, regarded as the most valuable forest habitats of the country.

### Presence-only maximum entropy modeling approach

**Fig 2:**
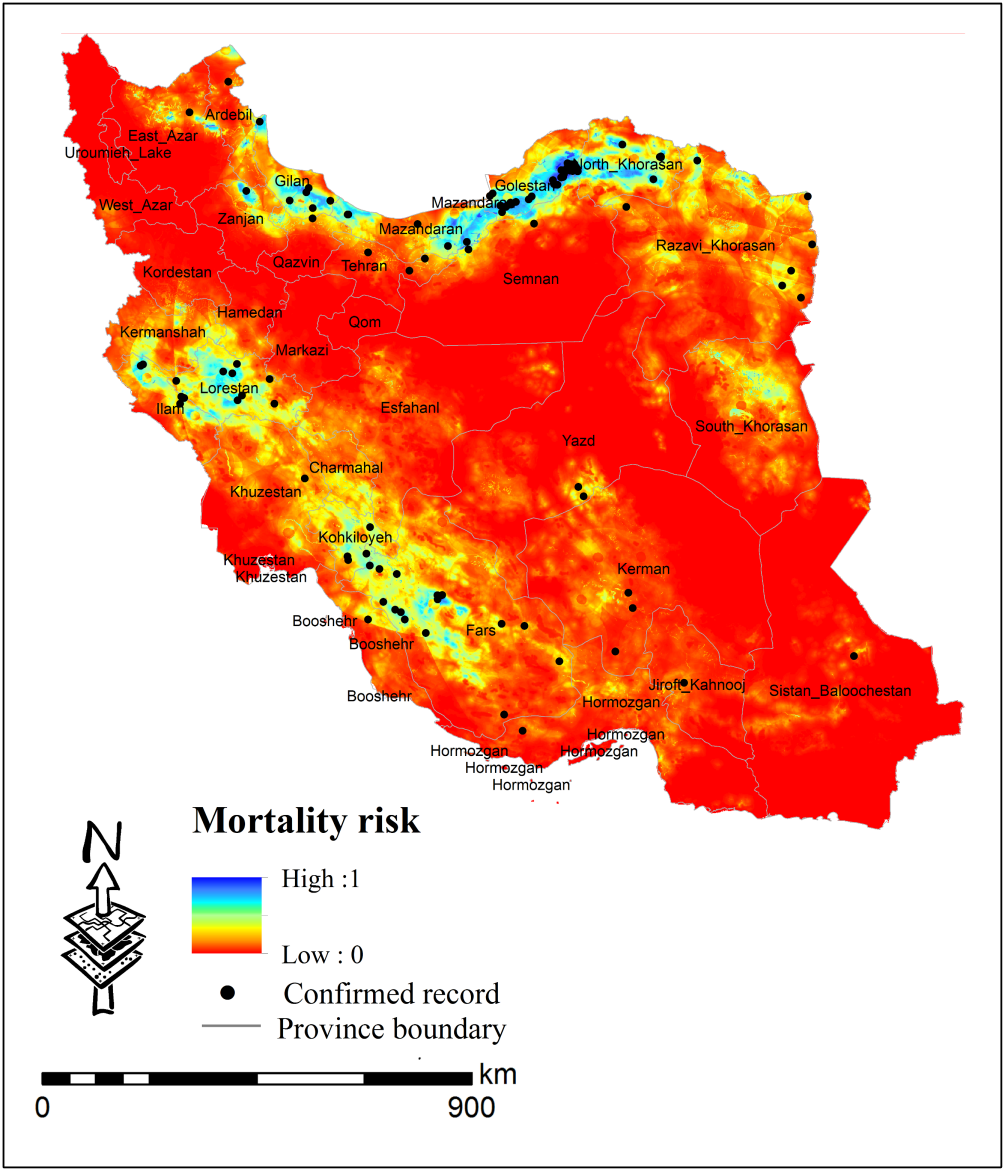
Persian leopard’s unnatural mortality risk map resulting from MaxEnt modeling approach.

The risk map of unnatural mortality of Persian leopard produced using the MaxEnt modeling approach, shows areas facing a high risk of unnatural mortality throughout the country (Fig 2).

**Fig 3:**
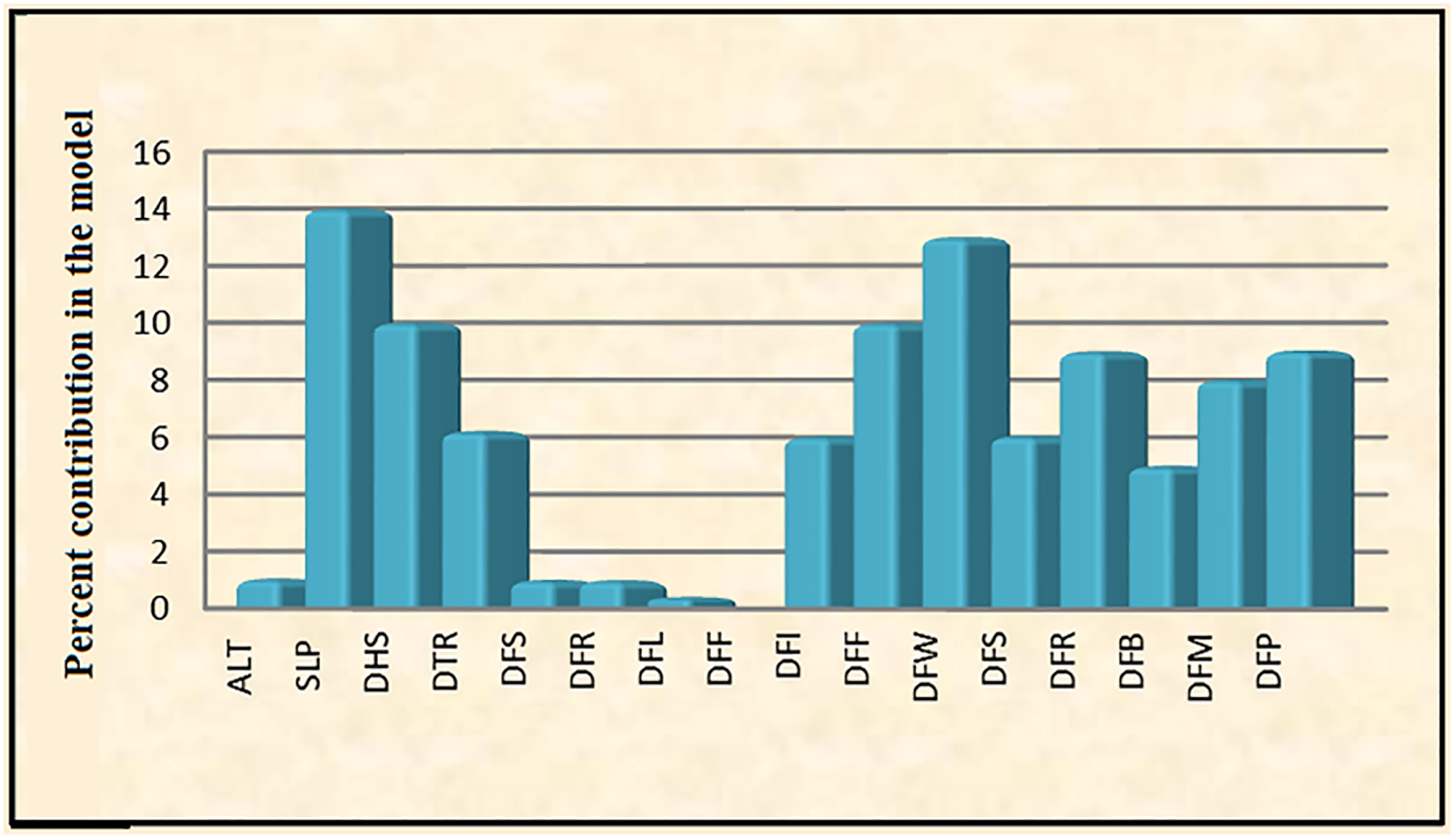
Habitat variables percent contribution in the model (ALT = Altitude, SLP = Slope, DHS = Distance to the human settlements, DTR = Distance from the traffic roads, DFS = Distance from streams, DFL = Distance from lakes, DFF = Distance from dry farming lands, DFI = Distance from irrigated farming lands, DFF = Distance from forested areas, DFW = Distance from woodlands, DFS = Distance from scrublands, DFR = Distance from range lands, DFB = Distance from bare lands, DFM = Distance from rocky mountainous areas, DFP = Distance from protected areas).

**Fig 4:**
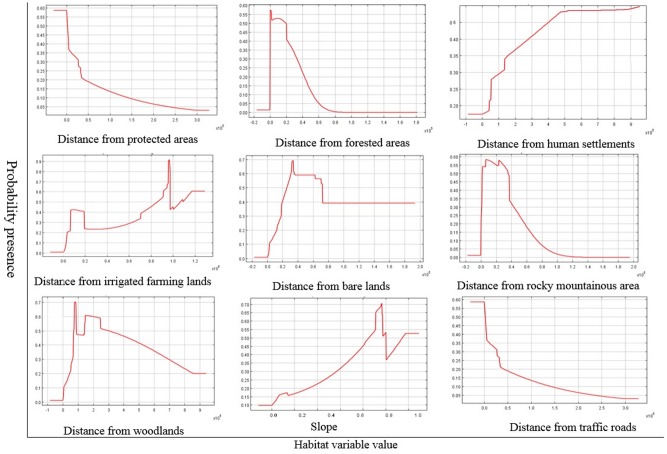
Response curves of MaxEnt modeling approach for Persian leopard unnatural mortality (only the most significant variables have been shown).

Based on the response curves generated by the model, we found that slope, a topographical variable, and the two distance variables of distance from woodlands and distance from human settlements were the main factors affecting species presence and activity at the recorded points (Figs 3 and 4).

According to this risk map, Persian leopards probably face a high risk of unnatural mortality in the north-eastern and south-western parts of Iran. Alternatively, unnatural mortality points represent frequent presence of the Persian leopard and hence can be regarded as its preferred macro or meso-habitat. Because the populations of leopards show a declining trend and recording presence points by field trips can be very time-consuming and expensive, the map generated by the model can function as a distribution map for the species.

## Discussion

We received unnatural mortality records from most of the provinces, which represents a larger area compared to Sanei et al report (18 provinces) [31, 32]. Shooting and deploying toxic baits result in severe reduction of natural prey items, leading to depredation on livestock by leopards. [8]. Higher unnatural mortality of males (about two times more than females), may be due to males’ larger home range, wider habitat usage and dispersion compared to females [33, 34].

Balm et al. [34] found that all dead leopards outside protected areas were male. In our study, we found that more than 84 percent of Persian leopards’ unnatural mortality caused by shooting and prey poisoning occurred outside protected areas. Such findings indicate that although protected areas themselves are relatively safe for leopards, the current coverage of Iranian protected areas is not sufficient to secure all the needs of such flagship species, leaving space for the expansion of present protected areas or the establishment of new ones. Including more buffer to the protected areas with less severe management intensity, e.g. no-hunting areas, can help the species. Similar findings have been reported by Taghdisi et al. [35] in which they found prey abundance and accessibility are the most important habitat variables affecting distribution and survival of leopards in Iran.

Swanepoel et al. [22] reported that a large proportion of female unnatural mortality was accidental (traffic accidents), while the major cause of unnatural mortality of males was deliberate removal of individuals in unprotected areas. Regarding the sex ratio of cases, we found that road accidents involving males were about 2.5 times more common. The same trend was observed for death by shooting and poisoning with males more than 1.5 more likely to be killed by these means than females. It should be noted that most of such incidents took place outside protected areas. Our findings indicate the failure of Iranian protected areas to meet habitat requirements of the Persian leopards. As indicated in Figs 3 and 4, leopards tend to get closer to the rural areas where they can find more available prey items. In many protected areas of the country, the abundance of big game such as wild sheep, wild goats and wild boars have decreased dramatically resulting in predator shift towards human populated areas [31, 33]. Such a situation has been reported for other carnivores such as wolves that concentrate on livestock in some areas especially in northwestern and central parts of Iran [33]. Since this problem cannot be managed in short time periods, it is very important to execute plans and hold workshops for increasing the local communities’ awareness about the value of wildlife while compensating the damages imposed to their livestock.

Recently, as a result of global warming and climate change, precipitation patterns have changed dramatically all over the country [36] and wildlife suffer from freshwater scarcity, especially in arid and semi-arid areas. Water scarcity has affected the survival of Persian leopard populations, as we found increasing evidence of leopards drowning in wells, or water containers located outside protected areas.

Based on our data analysis, we found that more than 60 percent of unnatural mortality occurred due to consuming preys poisoned by people, shooting by ranchers, trophy hunters and military forces. In some cases, leopards, which had been trapped in the rural areas, were shot by military forces, which need urgent consideration. We strongly urge for mutual negotiation and understanding between environmental protection agencies and local military services regarding such emergency cases. Military personnel should be informed that shooting such endangered animals could lead to serious consequences to the species viability. In such cases, there should be trained people from DOE who can use Anesthetic Gun to capture the live animal and relocate it to the protected areas. Road mortality was the second most frequent cause of unnatural mortality, responsible for more than 26 percent of the total mortality of the Persian leopards. Similar factors for Persian leopard unnatural mortality have been reported by other authors [6, 33] who believe that illegal hunting, conflicts with local residents, habitat degradation and the decrease in abundance of natural prey are the most important factors threatening the species’ viability in Iran. Decreasing prey abundance cannot be regarded as a human-induced unnatural mortality factor directly. Nevertheless, it exerts a tremendous effect by driving large carnivores toward villages and livestock husbandry areas, which in turn leads to unnatural mortality in most cases. Habitat fragmentation and loss [37] have also forced leopards and other wild animals to cross roads more often, increasing the probability of road mortality.

**Fig 5:**
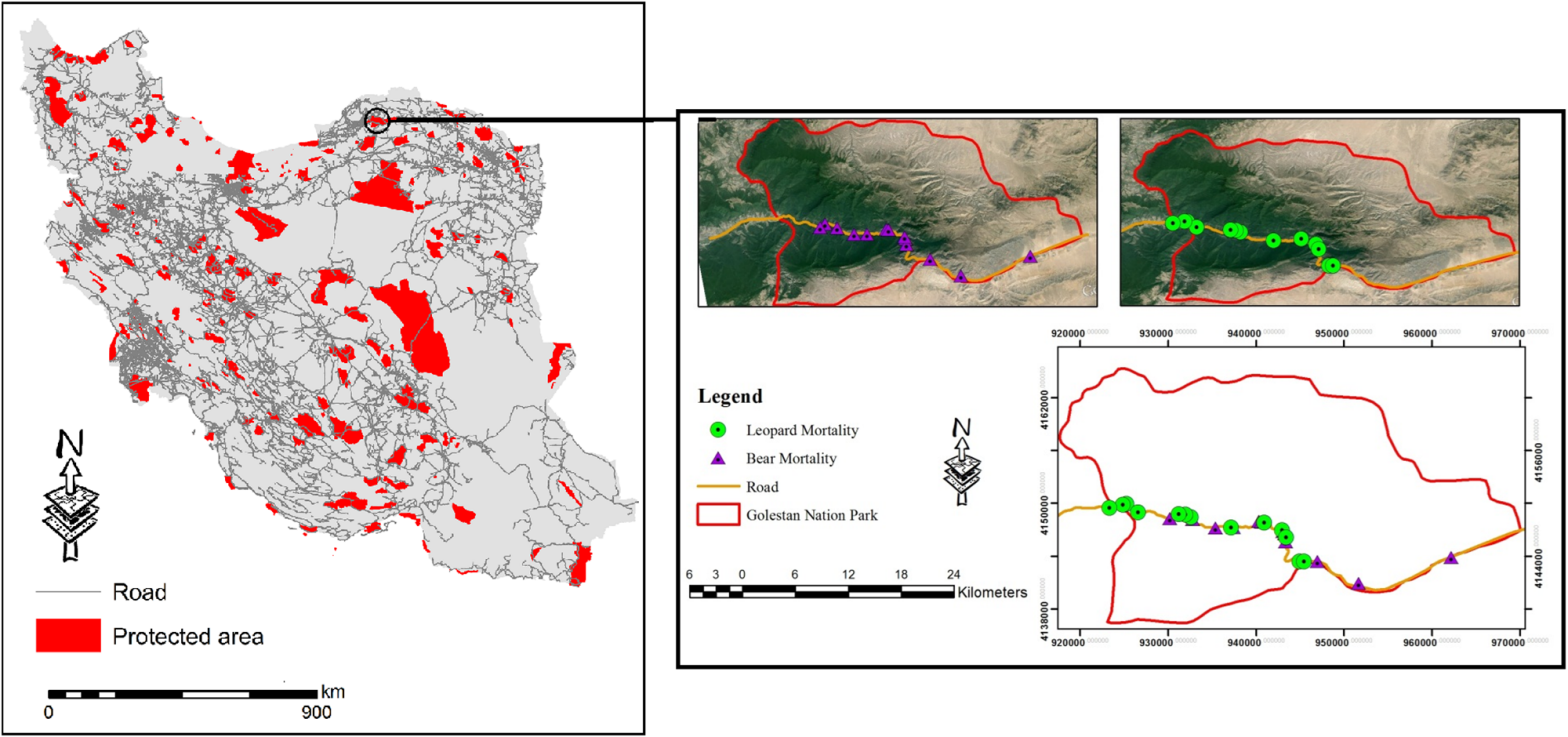
Persian leopard road mortality points in Golestan National Park which corresponds to the road mortality of brown bears (*Ursus arctos*). On the left, the intersection of the roads and highways with protected areas across the country has been shown.

Vehicle-animal collisions are a leading cause of Persian leopard unnatural mortality and have been reported frequently in Golestan National Park, the most important biodiversity hotspots in the heart of the Hyrcanian old-growth forests of the northern Iran. It was the first established protected area in Iran and was recognized as a reservoir in UNESCO MAB program in 1976 [38]. Unfortunately, more than 78 percent of Iran’s leopard collisions take place in this park, usually at the same places (Fig 5) where brown bears (*Ursus arctos*) suffer from road killings [39]. It is estimated that a considerable portion of leopard’s populations live in this park [6]; yet no wildlife crossings (faunal bridge) have been built there, in spite of one of the country’s busiest roads crossing through the park, connecting Tehran to Mashhad. Other species are also affected by the heavy traffic. An investigation showed that more than 117 foxes (*Vulpes vulpes*) and 262 jackals (*Canis aureus*) (based on data recorded for two decades from 1995 to 2015) had been killed on or along the roads, which cross Golestan National Park [39]. Our findings and maps can be useful in planning the construction of wildlife crossings in the park. We also recorded numerous accidents from five other areas, especially the Fars province (e.g. near southern border of Bamo National Park) located in southwestern Iran. Roads play an important role in habitat fragmentation and habitat destruction [37, 40–42] while simultaneously facilitating poachers’ and shepherds’ access to natural habitats [43]. Heavy-traffic roads cross more than 15 percent of protected areas in Iran (Naderi et al. unpublished data) which has presented new challenges to species with small and sparse populations such as large carnivores, especially leopards and cheetahs [3]. Regarding the first factor of the Persian leopard’s unnatural mortality, shooting and poisoning, we suggest that local villagers be educated about the value and importance of such species. Natives should be informed about the consequences of their illegal activities while outside organizations should compensate the damages imposed from leopards by depredation on livestock.

We can certainly claim that the development of roads in rural areas and especially in fragile mountainous regions without a complete environmental impact assessment is a new challenge threatening the population survival of leopards all over the country. As can be inferred from Fig 5, nearly all protected areas have been crossed by the traffic roads (highways and main roads) which need urgent consideration. Unfortunately, there is no faunal passage in such areas where could provide safe areas for wildlife to cross such infrastructures. Golestan National Park has high priority in constructing such bridges to prevent wildlife accidental mortality. It is necessary to promote educational opportunities as a way to increase rural and local people’s participation in environmental education and increase their awareness regarding the value and importance of wildlife. Locals should be aware of the benefits of protecting large carnivores. Meanwhile it is necessary that a mutual understanding to be developed between Department of Environment (DOE) and Iranian Traffic Police to apply more speed limitations in the protected areas and the roads which pass close to these areas, in addition to installing speed cameras and wildlife passage signs.

## Supporting information

S1 Table This file shows the exact UTM localities of the Persian leopard accidental mortalities in Iran. (CSV)

## References

[1] Shaffer ML. Minimum population sizes for species conservation. Bioscience, 1981;31: 131–134.

[2] Farhadinia MS, Nezami B, Mahdavi A, Hatami K. Photos of Persian leopard in Alborz Mountains, Iran. Cat News, 2007;46: 34–35.

[3] Gubbi S, Poornesha HC, Daithota A, Nagashettihalli H. Roads emerging as a critical threat to leopard in India? Cat News, 2014;60: 30–31.

[4] Drews C. Road kills of animals by public traffic in Mikumi National Park, Tanzania, with notes on baboon mortality, Afr J Ecol. 1995;33(2): 89–100.

[5] Baskaran N, Boominathan D. Road kill of animals by highway traffic in the tropical forests of Mudumalai Tiger Reserve, southern India. Jo TT. 2010;2(3): 753–759.

[6] Khaleghi MR, Ghoddusi J, Ahmadi H. Regional analysis using the Geomorphologic Instantaneous Unit Hydrograph (GIUH) method. SWR. 2014;9: 25–30.

[7] Swanepoel LH, Somers MJ, van Hoven W, Schiess-Meier M, Owen C, Snyman A et al Survival rates and causes of mortality of leopards *Panthera pardus* in southern Africa. Oryx, 2015b;49: 595–603.

[8] Nowell K, Jackson P. Wild cats Status survey and Conservation Action Plan: IUCN/SSC, Cat Specialist Group, IUCN, Gland, Switzerland; 1996.

[9] Kitchener A. The Natural history of the wild cats. Comstock Publishing Associates, Ithica, New York; 1991.

[10] Ghoddusi H. Dynamic Investment in Extraction Capacity of Exhaustible Resources. SJPE. 2010;57(3): 359–373

[11] Khorozyan I, Malkhasyan A, Asmaryan S. The Persian leopard prowls its way to survival. Endanger Species Update. 2005;22: 51–60.

[12] Rostro-Gracia S, Abadfe L, Tharchen L, Cushman S, Macdonald D. Scale dependence of felid predation risk: identifying predictors of livestock kills by tiger and leopard in Bhutan, Landsc Ecol. 2016;31(6): 1277–1298

[13] Erfanian B, Mirkarimi SH, Mahini AS, Rezaei HR. A presence-only habitat suitability model for Persian leopard *Panthera pardus saxicolor* in Golestan National Park, Iran, Wildlife Biol. 2013;19(2): 170–178.

[14] Treves A, Karanth KU. Human-Carnivore Conflict and Perspectives on Carnivore Management Worldwide. Conserv Biol. 2003;17: 1491–1499.

[15] Sunquist M., Sunquist F. Wild cats of the world. The university of Chicago press, 2002.

[16] Weaver JL, Paquet PC, Ruggiero LF. Resilience and conservation of large carnivores in the Rocky Mountains. Conserv Biol. 1996;10: 964–976.

[17] Andren H, Linnell JDC, Liberg O, Andersen R, Danell A., Karlsson J et al Survival rates and causes of mortality in Eurasian lynx (*Lynx lynx*) in multi-use landscapes. Biol Cons. 2006;131: 23–32.

[18] Dalerum F, Shults B, Kunkel K. Estimating sustainable harvest in wolverine populations using logistic regression. J Wildl Manage. 2008;72: 1125–1132.

[19] Swanepoel LH, Balme G, Williams S, Power RJ, Snyman A, Gaigher I et al A conservation assessment of *Panthera pardus* In Child MF, Roxburgh L, Do Linh San E, Raimondo D, Davies-Mostert HT, editors. The Red List of Mammals of South Africa, Swaziland and Lesotho. South African National Biodiversity Institute and Endangered Wildlife Trust, South Africa, 2016.

[20] Morten O, Wegge P. Spacing and activity patterns of leopards *Panthera pardus* in the Royal Bardia National Park, Nepal, Wildlife Biol. 2005;11(2): 145–152

[21] Krebs J, Lofroth E, Copeland JP, Banci V, Cooley D, Golden H et al Synthesis of survival rates and causes of mortality in North American wolverines. J Wildl Manage. 2004;68: 493–502.

[22] Swanepoel LH, Somers MJ, Dalerum F. Density of leopards *Panthera pardus* on protected and non-protected land in the Waterberg Biosphere, South Africa, Wildlife Biol. 2015a;21(5): 263–268

[23] Cheung WWL, Pitcher TJ, Pauly D. A fuzzy logic expert system to estimate intrinsic extinction vulnerabilities of marine fishes to fishing. Biol. Cons. 2005;124: 97–111.

[24] Zadeh L. Fuzzy sets. Information and Control, 1965; 8:338–353.

[25] Zadeh L.A. Fuzzy Logic. IEEE Computer, 1988;21: 83–92.

[26] Lin HX, Yen VC. Fuzzy sets and fuzzy decision-making, Boca Raton: CRC Press, 1995.

[27] Phillips SJ, Anderson RP, Schapire RE. Maximum entropy modeling of species geographic distributions. Ecol. Mod. 2006;190: 231–259

[28] Elith J, Phillips SJ, Hastie T, Dudík M, Chee YE, Yates CJ. A statistical explanation of MaxEnt for ecologists. Divers Distrib. 2011;17: 43–57.

[29] Fielding AH, Bell JF. A review of methods for the assessment of prediction errors in conservation presence/absence models. Environ Conserv. 1997;24(1): 38–49

[30] Swets JA. Measuring the Accuracy of Diagnostic Systems. Science, 1988;240: 1285–1293. 3287615 10.1126/science.3287615

[31] Sanei A, Mousavi M, Mousivand M, Zakaria M. Assessment of the Persian leopard mortality rate in Iran. UTM 11th International symposium on sustainability science and management. Terengganu, Malaysia, 2012.

[32] Kiabi BH, Dareshouri BF, Ghaemi RA, Jahanshahi M. Population status of the Persian leopard (*Panthera pardus saxicolor* Pocock, 1927) in Iran. Zool Middle East. 2002;25: 41–47.

[33] Swanepoel LH, Somers MJ, Van Hoven W, Schiess-Meier M, Owen C, Snyman A et al Survival rates and causes of mortality of leopards *Panthera pardus* in southern Africa. Oryx, 2015;49: 595–603.

[34] Balm G, Hunter L. Mortality in a protected leopard population, Phinda Private Game Reserve, South Africa: A population in decline? Ecolo. J. 2004;6: 1–6.

[35] Taghdisi M, Mohammadi A, Nourani E, Shokri S, Rezaei A, Kaboli M. Diet and habitat use of the endangered Persian leopard (*Panthera pardus saxicolor*) in northeastern Iran. Turk J Zool. 2013;7: 554–561.

[36] Ghafaripour S, Naderi M, Rezaei H. Investigating abundance, density and potential threats of Sand cat in the South-Eastern parts of Iran. JWB. 2017;1(1): 47–55.

[37] Ghoddusi A, Hamidi KHA, Ghadirian T, Ashayeri D, Moshiri H, Khorozyan I. The status of the Persian leopard in Bamu National Park, Iran. Cat News, 2008; 49: 10–13.

[38] Darvishsefat AA. Atlas of Protected Areas of Iran: Tehran, University of Tehran Press, 2006.

[39] Hemami MR, Saliari J, Esmaeili S. An investigation on pattern and diversity of wildlife mortality in the Golestan National Park, Environ. Res. 2016;7(14): 215–224 (in Persian)

[40] Treves A, Karanth KU. Human–carnivore conflict and perspectives on carnivore management worldwide Conserv Biol. 2003;17: 1491–1499.

[41] Hoskin CJ, Goosem MW. Road impacts on abundance, call traits, and body size of rainforest frogs in Northeast Australia. Ecol Soc. 2010;15(3), 15.

[42] Ree VDR, Jaeger JAG, Grift EAVD, Clevenger AP. Effect of roads and traffic on wildlife population and landscape function: Road ecology is moving toward larger scales. Ecol Soc. 2011; 16(1), 48.

[43] Switalski TA, Nelson CR. Efficacy of road removal for restoring wildlife habitat: Black bear in the Northern Rocky Mountains, USA. Elsevier, 2011;144: 2666–2673.

